# Undifferentiated epithelioid sarcoma presenting as a fever of unknown origin: a case report

**DOI:** 10.1186/s13256-018-1951-1

**Published:** 2019-01-27

**Authors:** Nicholas Sajko, Shannon Murphy, Allen Tran

**Affiliations:** 1 Clinical Research Centre, Dalhousie University, Room C-125, 5849 University Avenue, Halifax, NS B3H 4R2 Canada; 2 QEII Health Sciences Centre, Room 483 Bethune Building, 1276 South Park Street, Halifax, NS B3H 2Y9 Canada; 3 QEII Health Sciences Centre, Room 409 Bethune Building, 1276 South Park Street, Halifax, NS B3H 2Y9 Canada; 4 Dalhousie University, Faculty of Medicine, 1469 Oxford Street, Halifax, NS B3H 4R2 Canada

**Keywords:** Sarcoma, Fever of unknown origin (FUO), Complex, Rare, Soft tissue, Malignancy

## Abstract

**Background:**

Fever of unknown origin is often a diagnostic dilemma for clinicians due to its extremely broad differential. One of the rarer categories of disease causing fever of unknown origin is malignancies; of these, soft tissue sarcoma is one of the least common. Soft tissue sarcomas make up < 1% of all adult malignancies and often do not present with any systemic manifestations or neoplastic fevers.

**Case presentation:**

A 73-year-old Caucasian woman presented with a 2-week history of fever and profound fatigue. The only other symptom she endorsed was a transient history of left knee pain, initially thought to be unrelated. There was no clear cause on initial examination and routine investigations, but her C-reactive protein was significantly elevated at 207 mg/L. Blood cultures and a urine culture were drawn. She was admitted to hospital for further investigation and placed on empiric antibiotics. Her blood cultures were negative, but she had one further fever in hospital. Computed tomography scans did not yield a cause of her fever. No vegetations were seen on echocardiography. Antibiotics were stopped as she did not seem to have an acute infectious cause of her fever. No new symptoms developed. She felt well enough to proceed with out-patient follow up and was discharged after 8 days in hospital. At 1-month post-discharge: no resolution of symptoms, but she endorsed a recurrence of her left knee pain. Ultrasound and magnetic resonance imaging revealed a 4.5 × 6.8 × 11.6 cm soft tissue mass, identified as a sarcoma on biopsy. She subsequently underwent a distal femur resection. Final staging was pT2bN0M0. She underwent adjuvant radiation therapy, but was found to have developed metastatic disease.

**Conclusion:**

This case revealed an atypical presentation of a rare soft tissue sarcoma as the cause of the illness. The etiology behind a fever of unknown origin can be difficult to elucidate, making the approach to investigation particularly important. Repeated history-taking and serial physical examinations can be crucial in guiding investigations and ultimately arriving at a diagnosis. In addition, we believe this case highlights the adage that no seemingly innocuous symptom should be left out when working up a condition with such an extensive and complex differential.

## Key points

- Fever of unknown origin (FUO) commonly presents with atypical manifestations of the underlying cause. In these scenarios, no symptom should be deemed insignificant.
- Repeated examinations over time are often needed to elucidate the cause of FUO.
- FUO is an uncommon presentation for sarcoma but can occur.

## Introduction

The case described is of a patient with non-specific constitutional symptoms, an initial history of transient left knee pain, and the development of a FUO. Workup eventually led to the diagnosis of an undifferentiated sarcoma of epithelioid morphology. This diagnosis is not only rare in terms of sarcoma incidence rates, but also because of its unusual systemic presentation [1].

Sarcomas are a heterogeneous group of malignant tumors of mesenchymal origin, representing roughly < 1% of all adult malignancies. Approximately 90% of cases of sarcoma are defined as soft tissue sarcomas (STSs): developing in the muscles, deep tissues, blood vessels, nerves, fat, and joints, while the rest are classified as malignant bone tumors [2].

The Surveillance, Epidemiology, and End Results (SEER) database indicates that the incidence rates of STS development are highest in children < 5 years of age and in adults > 50-years old, with the lowest incidence found in young adults [2]. Numerous predisposing factors have been considered and include various genetic, environmental, and iatrogenic entities; however, no definitive or specific etiologies have been identified (Table 1) [2].

**Table 1:** Factors and exposures currently believed to be related to the development of various sarcoma subtypes

Risk factors	
Environmental exposure	Ionizing radiation exposure
	Phenoxyacetic acids
	Chlorophenol
	Vinyl chloride
	Dioxins
Infectious agents	HIV
	HHV8
Genetic conditions	Li–Fraumeni syndrome
	Neurofibromatosis type 1
	Retinoblastoma (13q14)
	Paget disease
	Werner syndrome
	Bloom syndrome
	Gardner syndrome

*HHV8* human herpesvirus 8, *HIV* human immunodeficiency virus

STS can be divided extensively by histologic subtypes, for which the World Health Organization (WHO) currently lists more than 100. These malignancies can also be described in terms of location and specific histologic morphology. Site distribution favors the lower extremities (29%), with tumors less often involving the upper extremities (11%) and trunk (10%) (Table 2) [3]. Morphology is often used as a descriptor for the undifferentiated/unclassified subtype, as was the case with our patient who was discovered to have an undifferentiated sarcoma of epithelioid morphology.

**Table 2:** Anatomic distribution of soft tissue sarcomas reported by the Memorial Sloan Kettering Cancer Center (MSKCC) between 1982 and 2011 (*n* = 9040)

Site distribution	
Lower extremities	29%
Visceral	22%
Retroperitoneum	16%
Other	12%
Upper extremities	11%
Trunk	10%

In 1961, Petersdorf and Beeson defined a FUO as consisting of the following three criteria [4]:

1. A temperature of > 38.3 °C on several occasions,
2. > 3 weeks’ duration of illness, and
3. a failure to reach a diagnosis despite 1 week of in-patient investigations.

This definition has changed numerous times over the years due to our better understanding of various fever-inducing conditions and improved diagnostic capabilities [5]. The most widely used definition now divides FUO into four major categories, each having specific definitions and potential causes (Table 3) [6]. The most common category of FUO, and the one relating closest to the case presentation, is the “Classical FUO” subtype. A review of 26 studies of FUO shows the common etiologies of this subtype to include: infections (36.2%), malignancies (12.8%), non-infectious inflammatory diseases (20.9%), miscellaneous (6.2%), and undiagnosed (23.7%) [5].

**Table 3:** Subtypes of fever of unknown origin as defined by Durack and Street (1991) [6]

FUO subtype	Definition	Major causes
Classical FUO	- Temperature of ≧ 38.3 °C - > 3 weeks’ duration of illness - Failure to reach diagnosis with 1 week of in-patient investigations	Malignancies, infections, inflammatory diseases (non-infectious)
Hospital acquired FUO	- Temperature of ≧ 38.3 °C - Patient hospitalized ≧ 24 hours, fever not present or incubating on admission - Evaluation for ≧ 3 days	HAIs, postoperative complications, drug-induced
Immunocompromised or neutropenic FUO	- Temperature of ≧ 38.3 °C - Absolute neutrophil count ≦ 500 per mm^3^ - Evaluation for ≧ 3 days	Various bacterial, viral, and fungal infections
HIV-related FUO	- Temperature of ≧ 38.3 °C - Out-patient duration > 4 weeks; > 3 days for in-patients - Confirmed HIV infection	HIV defining infections (*Mycobacteria*, CMV, *Toxoplasma*, *Cryptococcus*), lymphoma, IRIS

*CMV* cytomegalovirus, *FUO* fever of unknown origin, *HAIs* hospital acquired infections, *HIV* human immunodeficiency virus, *IRIS* immune reconstitution inflammatory syndrome. (Adapted from table presented in Hayakawa *et al*., 2012 [5])

## Case presentation

A 73-year-old Caucasian woman with a medical history significant only for hypertension, presented to our emergency department complaining of intermittent subjective fever, anorexia, weakness, and fatigue for 2 weeks. Her subjective fevers were occurring almost nightly, and she had associated night sweats. Her weight was stable. She had a persistent non-productive cough. There was no sore throat or rashes. Her review of systems was negative for any other current symptoms. Her only medication was enalapril. Her family history was non-contributory.

She had been previously assessed by her family doctor for the same symptoms 2 weeks prior to this presentation. Routine investigations were unrevealing. At that time, she had left knee pain that developed after a hike the previous month. X-rays of her knee and femur were unremarkable. Her pain resolved within a week. No therapeutic interventions were undertaken at that time.

She had no sick contacts, no sexual partners, and no insect or tick bites. She had no known exposure to tuberculosis. She travelled to the Channel Islands 3 months before presentation. She had no animal exposures. She denied any history of injection drug use.

On initial examination, she appeared non-toxic. Her vital signs included a temperature of 38.6 °C, a heart rate of 96 beats/minute, blood pressure of 130/65 mmHg, and oxygen saturation of 99% on room air. There were no rashes and no lymphadenopathy was present. There were no signs of hyperthyroidism and the thyroid itself was normal in size without any nodules. Her jugular venous pulse was 2 cm above the sternal angle. She had normal heart sounds with no extra sounds or murmurs. There were no stigmata of endocarditis. Her lungs were clear with equal breath sounds bilaterally. An abdominal examination revealed a soft and non-tender abdomen. There was no hepatosplenomegaly, jaundice, or asterixis. Examination of her knees did not reveal any redness, warmth, effusions, or pain. A screening neurologic examination demonstrated grossly normal cranial nerves, full strength bilaterally, and normal reflexes, tone, and coordination. She was admitted for further investigation for her fever of unclear cause. Empiric piperacillin-tazobactam and intravenously administered saline were started on admission as acute bacterial infection was in the differential diagnosis.

Table 4 displays the results of her laboratory investigations. A peripheral smear was unremarkable. Serum free light chains were normal. No monoclone was found on serum protein electrophoresis. Urine analysis was bland. Five sets of blood cultures, a urine culture, and Lyme serology were negative. A chest X-ray was normal. Computed tomography (CT) scans of her head, neck, chest, abdomen, and pelvis were all unremarkable. A transthoracic echocardiogram revealed a normal heart with no vegetations.

**Table 4:** Results of laboratory investigations conducted as part of the workup for the patient described

Test	Results
White blood count (4.5–11.0 × 10^9^/L)	11.9 × 10^9^/L
Hemoglobin (120–160 g/L)	125 g/L (decreased to 109 g/L by discharge)
Mean corpuscular volume (80.0–97.0 fL)	87.5 fL
Platelet count (150–350 × 10^9^/L)	373 × 10^9^/L
Sodium (136–145 mmol/L)	142 mmol/L
Potassium (3.4–5.0 mmol/L)	4.7 mmol/L
Ionized calcium (1.15–1.27 mmol/L)	1.16 mmol/L
Creatinine (49–90 μmol/L)	60 μmol/L
Alanine aminotransferase (0–44 U/L)	44 U/L
Aspartate aminotransferase (5–45 U/L)	37 U/L
Alkaline phosphatase (38–150 U/L)	179 U/L
Gamma glutamyltransferase (0–49 U/L)	84 U/L
Total bilirubin (0.0–20.4 μmol/L)	11.0 μmol/L
Albumin (35–50 g/L)	24 g/L
Random glucose (3.9–7.8 mmol/L)	5.5 mmol/L
C-reactive protein (0–7.99 mg/L)	153.81 mg/L
Ferritin (6.5–204.0 μg/L)	883.7 μg/L
Lactate dehydrogenase (120–230 U/L)	203 U/L
Thyroid-stimulating hormone (0.35–4.30 mIU/L)	0.84 mIU/L
Creatine kinase (30–200 U/L)	49 U/L
Antinuclear antibody Antineutrophilic cytoplasmic antibodies Anti-smooth muscle antibody Anti-mitochondrial antibody Rheumatoid factor	Below detectable limits

She had one further temperature of 39.4 °C while in hospital, without any clear infectious source. Once the blood cultures were known to be negative, piperacillin-tazobactam was stopped. There was an impression that her workup could be continued on an out-patient basis as immediately life-threatening causes of fever had been ruled out. She was discharged home after an 8-day admission in hospital with plan for out-patient follow up.

**Fig. 1:**
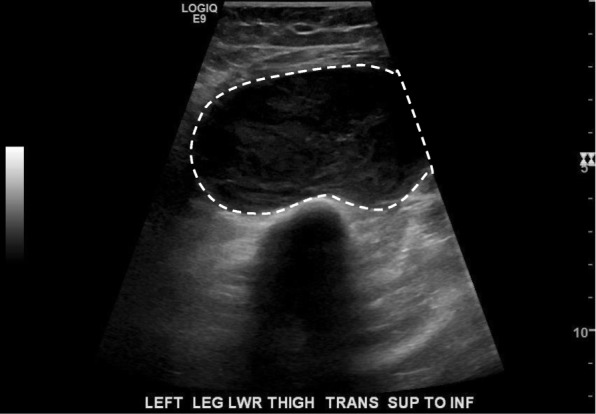
Ultrasound imaging of left thigh mass. Mass outlined with *dashed white line*

**Fig. 2:**
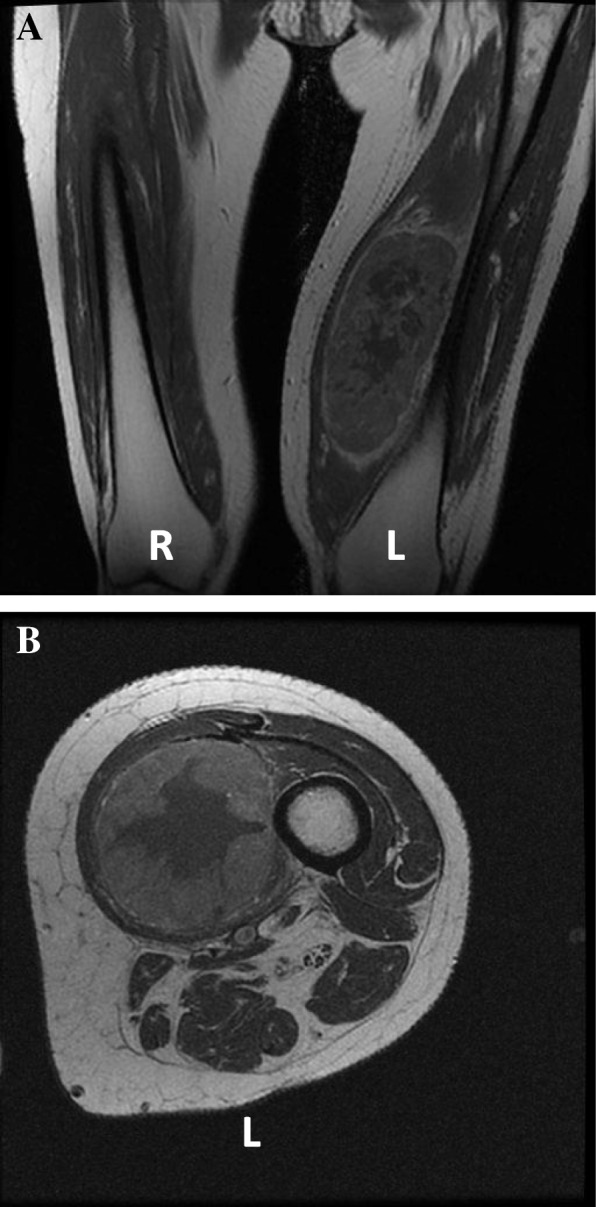
Magnetic resonance imaging investigation of left thigh mass. **a** Coronal magnetic resonance image of thighs. **b** Cross-sectional image of left thigh

She was seen 1 month after discharge. She had no improvement in her symptoms and noted a recurrence of her left leg pain. Her C-reactive protein (CRP) was 207 mg/L. On examination, she had a large, warm, left thigh mass. An urgent ultrasound revealed a 4.5 × 6.8 × 11.6 cm spindle-shaped, well-defined soft tissue mass with internal vascularity (Fig. 1). Magnetic resonance imaging (MRI) found that the mass met the femur but was not invading (Fig. 2). An initial biopsy revealed a poorly differentiated malignant neoplasm.

She underwent a distal femur excision with distal Global Modular Replacement System (GMRS) reconstruction. Final pathology revealed a grade 3, pT2bN0M0 undifferentiated sarcoma with epithelioid morphology. She had no nodal involvement or distant metastases at this time. Her CRP fell to 28.42 mg/L within 8 days of surgical excision. She recovered well from her surgery with resolution of her constitutional symptoms. She subsequently was planned to receive radiation therapy.

Prior to receiving radiation therapy, a follow-up CT scan was done a couple months after her surgery. This revealed the presence of a new 4 mm pulmonary nodule in the lower lobe of her left lung that was not felt to be a metastasis. There was no other evidence of distant metastases. Given these results, adjuvant radiation treatment was begun. She received 6600 cGy given in 33 fractions to her leg.

Roughly 1 month following the end of her radiation therapy course, she re-presented to our emergency room with painless hematuria and a month-long history of non-productive cough associated with decreased energy. CT scans of her chest revealed 16 pulmonary masses, measuring up to 6.2 cm. A CT scan of her abdomen and pelvis revealed a solitary nonobstructive renal calculus, as well as a new 3.2 × 6.5 cm pelvic mass.

She was subsequently referred to radiation and medical oncology where a shared decision was made to pursue palliative management.

**Fig. 3:**
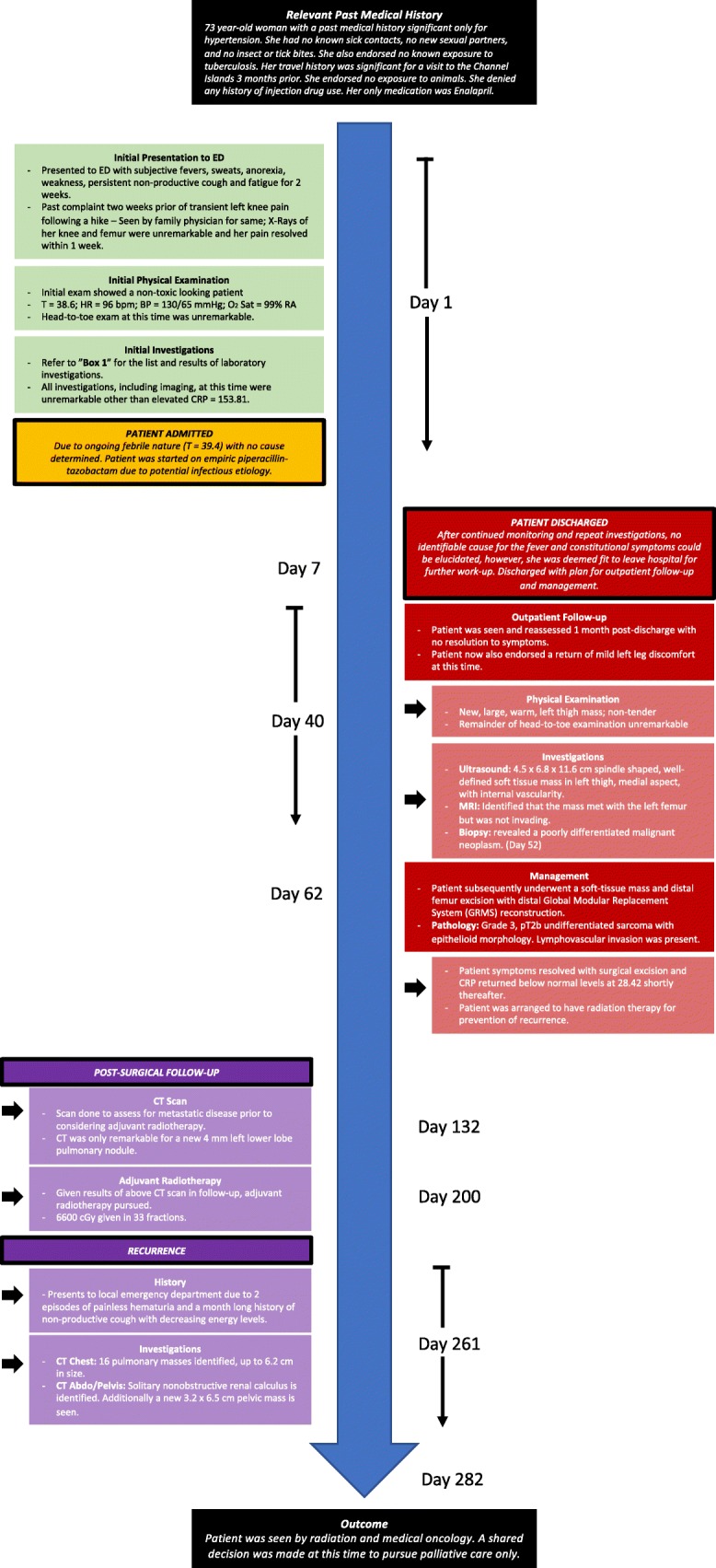
Case report timeline of events. *BP* blood pressure, *bpm* beats per minute, *CRP* C-reactive protein, *CT* computed tomography, *ED* emergency department, *HR* heart rate, *MRI* magnetic resonance imaging, *RA* room air, *Sat* saturation, *T* temperature

Figure 3 provides a timeline of the above described case.

## Discussion

The presence of FUO and neoplastic-based fevers with bone or STSs, as was the case with our patient, is not extensively reported in literature. In a cohort of 195 patients with bone and STSs who were admitted for treatment, 58 (30%) developed a fever while in hospital. Neoplastic fever was determined to be the cause in only 11 (6%) [1]. Due to the extensive number of other potential etiologies that could be responsible for FUO, the workup can be complex and extensive. An important part of this workup is obtaining a detailed history, as the major categories of classical FUO have distinct clinical hallmarks. Constitutional symptoms, anorexia, weight loss, fatigue, and malaise typify malignant/neoplastic etiologies of FUO. However, these vague symptoms need to be correlated to physical examination findings, laboratory studies, and diagnostic imaging, to make a definitive diagnosis. A retrospective review of 383 patients found that the mean time to diagnosis of a neoplastic cause of FUO after admission was 15.7 days [7]. This case, where extensive workup involved both in-patient and out-patient investigations lasting over 1 month, highlights the importance of a detailed history and repeated examinations when evaluating FUO. This patient initially had a history of transient left knee pain, which was previously investigated with only X-rays. When symptoms recurred, a focused physical examination of the area, as well as additional imaging studies, led to the diagnosis. Time to diagnosis may have been shortened should this history of knee pain been investigated earlier.

Investigation of FUO, guided by patient history and physical examination findings, should begin with a broad systematic approach to narrow the differential as efficiently as possible. Table 5 outlines a practical approach for the initial workup of FUO. Imaging techniques, such as fluorine-18-fluorodeoxyglucose (FDG) positron emission tomography (PET)/CT, play an increasingly important role in the investigation of FUO without any diagnostic clues. One review found FDG-PET/CT contributed to determining the final causal diagnosis in FUO in 38–75% of cases [8]. In addition, a systematic review examining data from 16 trials found that PET/CT imaging was 90% sensitive and 89% specific for overall diagnostic accuracy in osseous and STSs [9]. However, this imaging modality was not utilized in this case. Whether or not its use would have led to an earlier diagnosis is something that could be considered given the above data.

**Table 5:** Initial investigations in classical fever of unknown origin

Diagnostic test	Potential diagnostic utility
Initial investigations	
CBC with differential and smear	- Neutropenia to determine breadth of possible infection, cytopenias suggesting bone marrow involvement
Renal function	- Glomerulonephritis
Liver enzymes and LFTs	- Hepatic or cholestatic etiologies
ESR and CRP	- Markers signifying an inflammatory process
Thyroid function	- Thyrotoxicosis
HIV antibody	- HIV and potential for opportunistic infections
Blood cultures	- Bacteremia or fungemia
Urine analysis and microscopy	- UTI, glomerulonephritis, or vasculitis
CXR	- Pneumonia, miliary TB, sarcoidosis, or malignancy
Investigations based on diagnostic clues from initial workup	
Echocardiography	- Potential endocarditis
CT	- Abscesses, other infections, or malignancy
Indium-111-WBC scan	- To localize any sites of inflammation
FDG-PET/CT	- Careful localization of infection, malignancy, vasculitis, or sarcoidosis
Lymph node biopsy	- Malignancy or infection
Liver biopsy	- Malignancy, infection, or autoimmune diseases
Bone marrow biopsy	- Hematologic malignancy
Temporal artery biopsy	- Giant cell arteritis

*CBC* complete blood count, *CRP* C-reactive protein, *CT* computed tomography, *CXR* chest X-ray, *ESR* erythrocyte sedimentation rate, *FDG* fluorine-18-fluorodeoxyglucose, *HIV* human immunodeficiency virus, *LFT* liver function test, *PET* positron emission tomography, *TB* tuberculosis, *UTI* urinary tract infection, *WBC* white blood cell

Prognosis is variable in FUO, reflecting the multitude of potential etiologies [5]. Overall, 12–35% of patients die from FUO-related causes, with malignant etiologies having the highest 5-year mortality at 52–100% [5]. This sharply contrasts with the 5-year mortality rate of 3.2% in those patients who remain undiagnosed after an extensive evaluation. Spontaneous recovery from FUO can occur in 51–100% of patients [5].

In the current era, limb sparing treatment procedures are often the preferred management option for STS. Along with surgical resection, adjuvant radiotherapy and chemotherapy can also be a key component to the management of STS. As was the case with our patient who had a grade 3 STS, most moderate to high-grade STSs receive adjuvant radiotherapy. The combination of local resection and radiotherapy has similar survival and recurrence rates as amputation, but with the added benefit of functional preservation [10]. However, the role for adjuvant chemotherapy is more controversial and is dependent on the histologic subtype and morphology of the tumor, as well as the presence of metastases [10]. Although our patient did develop metastatic disease, she had opted to pursue palliative care in lieu of further aggressive treatment.

## Conclusion

In conclusion, determining an etiology in a patient with FUO can be challenging due to the breadth of possibilities. It is not only challenging, but FUO often represents an atypical presentation of a disease. This case provides such an example and aims to highlight the extensive workup required. In addition, it supports the adage that a full detailed history and examination is often a necessity when evaluating FUO; repeated history and examinations may be needed, with no symptom being viewed as irrelevant. Findings then must be used to direct further investigations. An investigatory approach is outlined in this case report and aims to provide clinicians a foundation from which to work from in the workup of similar cases.

## Abbreviations

CRP: C-reactive protein
CT: Computed tomography
FDG: Fluorine-18-fluorodeoxyglucose
FUO: Fever of unknown origin
GMRS: Global Modular Replacement System
MRI: Magnetic resonance imaging
PET: Positron emission tomography
SEER: Surveillance, Epidemiology, and End Results
STS: Soft tissue sarcoma
WHO: World Health Organization
